# Supplementary material for: Genetic Variability in Carotenoid Contents in a Panel of Genebank Accessions of Temperate Maize from Southeast Europe
Source: Plants (Basel). 2023 Sep 30;12(19):3453. doi: 10.3390/plants12193453 (PMC10575074; doi:10.3390/plants12193453)
Supplement: Supplementary file 1 [file plants-12-03453-s001.zip › plants-2619872-supplementary.pdf]

Table S1. Maize accessions from Croatian plant genetic resources database (CPGRD) used in the study, their parentage, percentage of native germplasm, color and type of kernel

No.	CPGRD code	Accession name	Background	% native germplasm	Kernel color†	Kernel type‡
1	CAM00059	LTW109-2	LF x(L131F-d x LE)	23	O	F
2	CAM00060	LTW112	(L1-26 x SD36)x SD36	23	Y	SF
3	CAM00061	LTW126	(Sin39 x Sin38)x B88	0	O	F
4	CAM00062	LTW21	L86 x(Bc488 x L85)	75	O	SD
5	CAM00063	LTW32-2	H99 x(L131F-d x H99)	23	O	SD
6	CAM00064	LTW56-2	L90 x(B70 x H99)	50	O	F
7	CAM00065	LTW60	(SD36 x H99)x L81ŠL	44	Y	SD
8	CAM00066	LTW63	L86 x(Bc488 x LF)	50	Y	SF
9	CAM00067	LTW8-1	L80 x(D-Bc7-9549 x D-Bc7-608)	50	Y	SD
10	CAM00068	LTW84-1	SD36 x(L80 x SD36)	23	PY	SD
11	CAM00069	LTW85	L81ŠL x(SD36 x H99)	44	O	F
12	CAM00070	TH126	SD36 x Pa402	0	O	SD
13	CAM00071	TH 13-3	L86 x SD36	50	O	SD
14	CAM00072	TH136	L1-26 x L131F-d	94	O	F
15	CAM00073	TH14-1	L86 x SD36	50	Y	SD
16	CAM00074	TH152	L81/ZMR x SD36	50	Y	SD
17	CAM00075	TH158	L131F-e x SD36	47	Y	SF
18	CAM00076	TH163	L1-26 x SD36	47	O	SD
19	CAM00077	TH167	Lady Finger x Long Ear material	0	Y	SD
20	CAM00078	TH174	L1-26 x L131F	100	O	SF
21	CAM00079	TH18-2	L86 x L131F-d	97	O	F
22	CAM00080	TH21	L86 x L131F-d	97	O	SD
23	CAM00081	TH229-2	L131F-e x BSTL	47	PY	SF
24	CAM00082	TH249	L219 x red stalk material	50	DO	SD
25	CAM00083	TH251	H99 x B88	0	O	SD
26	CAM00084	TH288-3	Pa402 x SD36	0	Y	SF
27	CAM00085	TH289	Pa402 x SD36	0	O	SF
28	CAM00086	TH295-1	L80 x SD36	50	PY	D
29	CAM00087	TH30-2	L1-26 x L131F	100	Y	D
30	CAM00088	TH304-1	L131F-e x SD36	47	O	F
31	CAM00089	TH322	L81ŠL x LE (long ear material)	44	Y	F
32	CAM00090	TH348	L131F-d x SD36	47	Y	SF
33	CAM00091	TH350-2	L131F x LF (lady finger)	50	Y	SF
34	CAM00092	TH351	L1-26 x BSUL	47	Y	SD
35	CAM00093	TH352	L1-26 x BSUL	47	Y	SF
36	CAM00094	TH367	B87 x H99	0	O	F
37	CAM00095	TH369-1	B87 x H99	0	DO	SF
38	CAM00096	TH370	L131F-d x Mich80-3	47	DO	SF

39	CAM00097	TH374-1	L131F-d x B88	47	O	SD
40	CAM00098	TH378-1	L131F-e x MICH (unknown material from Michigan Univ.)	47	O	F
41	CAM00099	TH381	L86 x Mo17	50	Y	SD
42	CAM00100	TH384	L86 x SD36	50	Y	SF
43	CAM00101	TH387	L81ŠL x red stalk material	44	Y	F
44	CAM00102	TH388-2	L80 x Mo17	50	Y	SF
45	CAM00103	TH390-3	L80 x Mo17	50	O	SF
46	CAM00104	TH392-2	SD36 x B88	0	Y	SF
47	CAM00105	TH393-1	SD36 x B88	0	O	SF
48	CAM00106	TH410-1	SD36 x L81ŠL	44	O	SD
49	CAM00107	TH411-1	SD36 x KAVA (material with coffee-like colour of grain)	0	O	F
50	CAM00108	TH42-1	Bc488 x L86	50	O	SF
51	CAM00109	TH421-1	SD36 x H99	0	O	SF
52	CAM00110	TH423	SD36 x Pa402	0	O	F
53	CAM00111	TH438	B70 x H99	0	O	SD
54	CAM00112	TH443-2	Bc24 x BSTL	0	O	SD
55	CAM00113	TH73-1	R588 x R59	0	Y	F
56	CAM00114	TH82-1	L86 x H99	50	O	SF
57	CAM00115	TH91-3	L86 x Pa402	50	O	SF
58	CAM00116	TH93-1	L86 x Pa402	50	O	SF
59	CAM00117	TH95	L80 x SD36	50	O	SF
60	CAM00118	TH128	SD36xPa402	0	Y	SD
61	CAM00119	TH143	R588xR59	0	Y	SD
62	CAM00120	TH148	L131F-d x SD36	47	O	SD
63	CAM00121	TH273-2	L131F-dxH99	47	O	F
64	CAM00122	TH275	SD36xPa402	0	Y	SD
65	CAM00123	TH282	L1-26xSE (short ear material)	47	O	SF
66	CAM00124	TH400	L131F-d x B88	47	Y	F
67	CAM00125	L80	Bujstina, population from Istra	100	O	F
68	CAM00126	10763	Unknown, Rugvica A41 (?)	0	O	SF
69	CAM00127	F7	Pop. Lacaune (France)	0	PY	F
70	CAM00128	H99-5	Illinois Syn.60C (Purdue)	0	DO	SF
71	CAM00129	LFB-3	"Šidski zuban", (L12 x Lady finger)x L12, 4x	97	O	SD
72	CAM00130	LFC-2	"Šidski zuban", (L12 x Lady finger)x L12, 4x	97	O	SD
73	CAM00131	LFC-2e	"Šidski zuban", (L12 x Lady finger)x L12, 4x	97	Y	SD
74	CAM00132	LM23-1	(L131F x L80) x (L131F x M/Tr)	75	O	F
75	CAM00133	LCA	Unknown (red aleurone)	0	Y	SF

76	CAM00134	LS39-5	Russian synth.(Sin.39 x L131F-d)	47	Y	SF
77	CAM00135	LE	Long ear material	0	O	F
78	CAM00136	LS34-2	Russian synth.(Sin.34)	0	PY	SD
79	CAM00137	LS36b	Russsian synth. (Sin.36)	0	PY	SD
80	CAM00138	NF	"Novosadski Fleischman"	100	Y	SF
81	CAM00139	TH240	LF x L81ŠL	44	O	SF
82	CAM00140	LTW119-1	H99 x(MICH x H99)	0	O	F
83	CAM00141	Pa402-1	USA, Pa402p Pioneer Syn.(S.African Syn.)	0	Y	SD
84	CAM00142	Pa402-2	USA, Pa402p Pioneer Syn.(S.African Syn.)	0	Y	SD
85	CAM00143	L68R-3	Obtained from Bc Institute, Rugvica, unknown	0	O	F
86	CAM00144	L5R-1	Obtained from Bc Institute, Rugvica, unknown	0	PY	D
87	CAM00145	L29R-1	Obtained from Bc Institute, Rugvica, unknown	0	O	SD
88	CAM00146	TZ41-1	Population from Tuzla, altitude 500m	100	O	SD

† PY-pale yellow; Y-yellow; O-orange; DO-deep orange
‡ D-dent; SD-semi dent; SF-semi flint; F-flint
